# ABC Transporters and Their Role in the Neoadjuvant Treatment of Esophageal Cancer

**DOI:** 10.3390/ijms19030868

**Published:** 2018-03-15

**Authors:** David Vrana, Viktor Hlavac, Veronika Brynychova, Radka Vaclavikova, Cestmir Neoral, Jiri Vrba, Rene Aujesky, Marcel Matzenauer, Bohuslav Melichar, Pavel Soucek

**Affiliations:** 1 Department of Oncology, Faculty of Medicine and Dentistry, Palacky University, Hnevotinska 976/3, 77515 Olomouc, Czech Republic; Marcel.Matzenauer01@upol.cz (M.M.); bohuslav.melichar@upol.cz (B.M.); 2 Biomedical Center, Faculty of Medicine in Pilsen, Charles University, Alej Svobody 76, 32300 Pilsen, Czech Republic; viktor.hlavac@lfp.cuni.cz (V.H.); veronika.brynychova@lfp.cuni.cz (V.B.); radka.vaclavikova@lfp.cuni.cz (R.V.); pavel.soucek@lfp.cuni.cz (P.S.); 3 Department of Surgery, Faculty of Medicine and Dentistry, Palacky University, Hnevotinska 976/3, 77515 Olomouc, Czech Republic; neoralc@fnol.cz (C.N.); j.vrba@upol.cz (J.V.); rene.aujesky@fnol.cz (R.A.); 4 Department of Surgery, Faculty Hospital Pilsen, Alej Svobody 80, 30460 Pilsen, Czech Republic

**Keywords:** esophagus, cancer, ABC transporters, chemotherapy, radiotherapy, prognosis, biomarker

## Abstract

The prognosis of esophageal cancer (EC) is poor, despite considerable effort of both experimental scientists and clinicians. The tri-modality treatment consisting of neoadjuvant chemoradiation followed by surgery has remained the gold standard over decades, unfortunately, without significant progress in recent years. Suitable prognostic factors indicating which patients will benefit from this tri-modality treatment are missing. Some patients rapidly progress on the neoadjuvant chemoradiotherapy, which is thus useless and sometimes even harmful. At the same time, other patients achieve complete remission on neoadjuvant chemoradiotherapy and subsequent surgery may increase their risk of morbidity and mortality. The prognosis of patients ranges from excellent to extremely poor. Considering these differences, the role of drug metabolizing enzymes and transporters, among other factors, in the EC response to chemotherapy may be more important compared, for example, with pancreatic cancer where all patients progress on chemotherapy regardless of the treatment or disease stage. This review surveys published literature describing the potential role of ATP-binding cassette transporters, the genetic polymorphisms, epigenetic regulations, and phenotypic changes in the prognosis and therapy of EC. The review provides knowledge base for further research of potential predictive biomarkers that will allow the stratification of patients into defined groups for optimal therapeutic outcome.

## 1. Introduction

The incidence of esophageal cancer (C15, EC) in the United States was 4.1 patients per 100,000 in 2010–2014 with the mortality curve almost copying the incidence curve, clearly indicating the disappointing treatment results [1]. EC manifests by the two distinctive histologic subtypes, esophageal squamous cell carcinoma (ESCC) and adenocarcinoma (EAC). ESCC is more common in Eastern Asia, linked mainly to dietary habits as intake of fruits, vegetables, antioxidants, meat, fat, smoking, and alcohol consumption [2] and its premalignant lesion is squamous dysplasia. EAC is frequent among Caucasians, associates with obesity and represents a stepwise transformation from gastroesophageal reflux disease (GERD), Barrett’s esophagus (BE) metaplasia, low-grade dysplasia, to high-grade dysplasia [3]. Despite years of experience, the treatment strategy has not changed significantly over the last decades and is based on studies conducted in the 1980s or 1990s [4,5]. Targeted therapy, which totally changed the patient prognosis in many tumor types, unfortunately has not yet brought substantial progress in the EC treatment. Similarly, new radiotherapy techniques such as stereotactic radiotherapy or proton beam therapy have also not improved patient outcome [6].

The generally recommended standard treatment strategy for localized (non-metastatic) EC is neoadjuvant chemoradiation followed by surgical resection (esophagectomy). However, considering the frequent poor performance status of the patients, this tri-modality technique is not feasible in all affected patients. At the same time, there is a subgroup of patients who develop metastases early after the treatment, and, obviously, the surgery, which significantly increases the morbidity and mortality of the treatment, does not improve and may even worsen patient outcome. At present, we do not have any predictive biomarkers for tumor resistance to chemotherapy and radiotherapy which could potentially identify the subgroup of patients in whom the treatment should be less aggressive and should rather comprise palliative and supportive care. At the same time, a subgroup of patients who may achieve complete response after the neoadjuvant chemoradiation exists, and the surgical resection in these patients could be potentially omitted without affecting the outcome. This omission of esophagectomy may significantly shorten the treatment course and recovery after neoadjuvant chemoradiation and, therefore, dramatically reduce the cost of the whole treatment process and improve the patient quality of life.

Considering the fact that the response to chemotherapy and radiotherapy ranges from excellent response, i.e., patients achieving complete response and long-term survival, to rapid progression even during neoadjuvant treatment, the role of molecular factors contributing to these scenarios requires full attention.

Development of resistance to chemotherapy is a multifactorial process, which is mediated by dysregulated apoptosis and autophagy, enhanced DNA repair, mitochondrial alterations including deregulation of oxidative stress production, epithelial–mesenchymal transition and cancer cell stemness, inactivation of drugs by biotransformation enzymes, and by disequilibrium between drug efflux and uptake due to alterations in expression and activity of membrane transporters [7]. This review focuses on adenosine triphosphate (ATP)-binding cassette (ABC) transporters whose importance has already been studied in several cancers [8,9,10,11]. Biomarkers play a critical role in the management of cancer patients [12], and discovery and validation of reliable predictive biomarkers should improve clinical approaches to treat EC and provide a further step towards personalized medicine.

**Figure 1:**
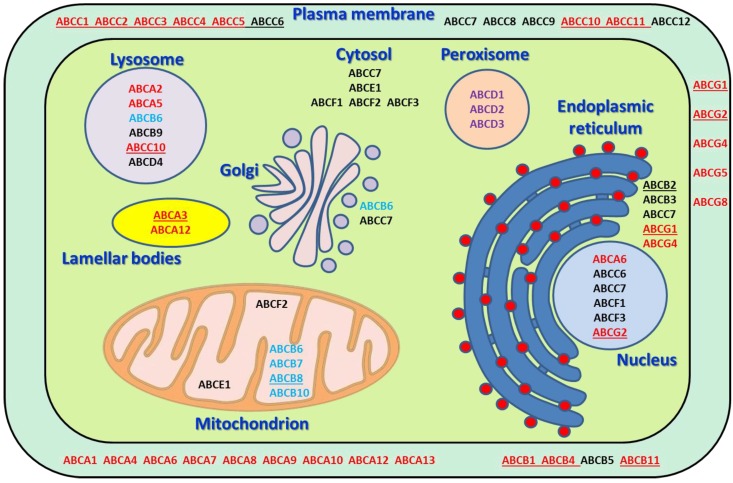
Schematic depiction of localization of ABC transporters within the cell and their major functions, with phospholipid, sterol and bile salts transporting ABCs in red; heme transporters in cyan; fatty acid transporters in violet; and ABCs with specific functions in black. Anticancer drugs transporters are underlined.

ABC transporters represent a group of membrane transporters which translocate different molecules through the cellular membrane mostly to extracellular space (except several compartment-specific transporters) using the ATP as an energy source (Table 1 and Figure 1). ABCs can be divided into several subgroups named A–G according to the amino acid similarity. Forty-eight genes coding functional ABC transporters and one pseudogene (*ABCC13*, OMIM: 608835) have been identified in humans [13].

**Figure 2:**
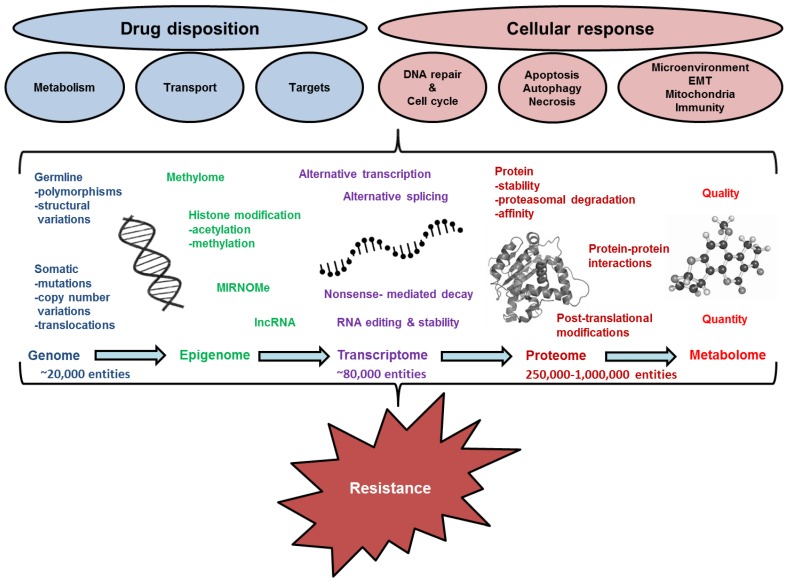
Schematic diagram of pathways and mechanisms of resistance. EMT, epithelial-to-mesenchymal transition.

As in other proteins, several mechanisms are involved in the alteration of activity (phenotype) of ABC transporters, including: germline genetic background, e.g., single nucleotide polymorphisms (SNPs) and structural variations; somatic genetic aberrations, e.g., mutations, copy number variations and translocations; and epigenetic changes such as DNA methylation, histone acetylation/deacetylation or deregulation of microRNA expression. These events lead to dysregulation of gene transcription (mRNA level), aberrant transcript splicing or stability and finally may result in changes of protein level, stability, and activity. Posttranslational processing complements all above processes and may also be driven both genetically and epigenetically. All these mechanisms are, therefore, investigated as potential source of causal or correlative biomarkers of carcinogenesis and tumor progression, including chemoresistance (see Figure 2). Both correlative and causal biomarkers may be used for estimation of prognosis of cancer patients and for prediction of optimal therapy regimens. Furthermore, causal biomarkers can further be exploited as targets for design of novel treatments. Finally, the era of liquid biopsy brought another interesting source of biomarkers in the patients’ blood circulation where traces of tumor-related genetic and epigenetic factors may be followed in the course of therapeutic process and used for monitoring of therapy efficacy, tumor relapse or extent of minimal residual disease.

This review aims to provide first knowledge base of all aspects covering biomarker research in the area of ABC transporters related to EC with special regard to the neoadjuvant setting. Highlighting promising molecular targets which could predict the chemotherapy and/or radiotherapy resistance represents a major goal of this paper. With help of such biomarkers, patients could be divided into subgroups with either high chance for good response to the treatment where surgery may be avoided or patients with predicted poor response where symptomatic care should be applied to avoid the exposure of the patients to ineffective treatment.

## 2. Search Results and Discussion

**Figure 3:**
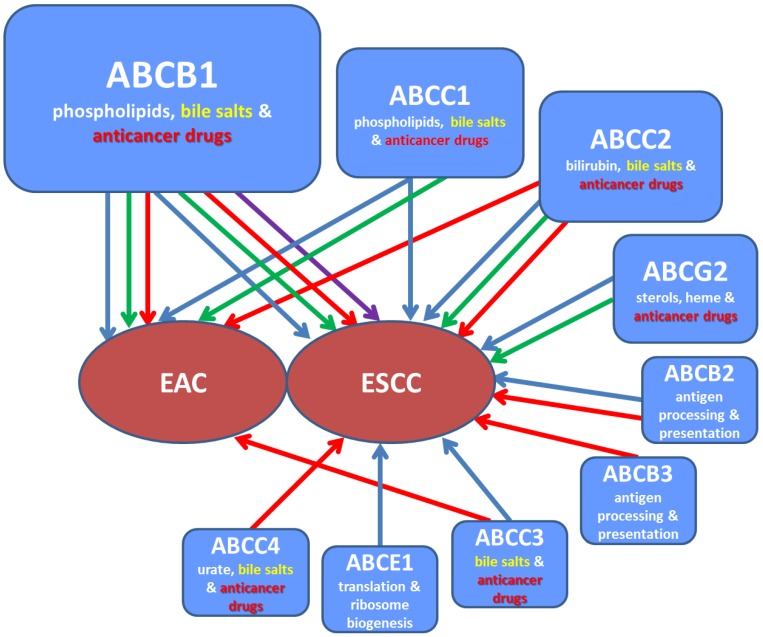
Schematic overview of associations between ABC transporters and risk or progression of EC. Associations are symbolized by arrows (blue for protein, green for transcript, red for gene, and violet for miRNA). ABC transporters are shown in rectangles together with their major substrates (bile salts in yellow and anticancer drugs in red). EAC, esophageal adenocarcinoma; ESCC, esophageal squamous cell carcinoma.

Most relevant studies are summarized in Table 2, generalized in graphical form (Figure 3), and discussed in the text below. The text is divided to sections describing various aspects of dysregulation mechanisms from the general view to that connected specifically with ABC transporters.

### 2.1. Genetic Variation

Genetic variation can be divided into germline and somatic changes with SNPs being the most frequently studied germline variants. Structural variations on the other hand represent larger changes in genome, e.g., copy number variations (CNV) as deletions or amplifications, inversions, and translocations.

#### 2.1.1. Single Nucleotide Polymorphisms

SNPs in ABC transporters can significantly change the activity. Narumiya et al. have reported that SNP rs1045642 in *ABCB1* (alias Pgp or MDR1, 171050) gene may affect the patient response to neoadjuvant chemoradiation, and, more specifically, the response of lymph node invaded by tumor cells [14].

The above-mentioned SNP rs1045642 is a silent substitution in codon 3435 (C3435T) of the *ABCB1* gene and T allele seems responsible for slower metabolism of some drugs and TT genotype is suspected to moderately increase risk for certain cancers [15]. On the other hand, the T allele might bring an advantage for patients treated with drugs that are substrates of *ABCB1*. rs1045642 has been mentioned in several studies focused on EC. A study of rs3213619 (T-129C, promoter region), rs1045642, rs1128503 (C1236T) and rs2032582 (Ala892Ser) SNPs in *ABCB1* gene observed that haplotypes composed of these SNPs predict risk of ESCC or colorectal cancer, although these trends were not significant [16]. Subsequently, a study involving 210 ESCC patients treated with neoadjuvant chemoradiation, based on cisplatin, 5-fluorouracil, or paclitaxel, followed SNPs rs2032582 and rs1045642 in *ABCB1*. Allele T in rs1045642 was significantly associated with reduced hazard risk of death and with an improved overall survival in patients treated with cisplatin (*n =* 146) [17]. The rs2032582 SNP has recently been studied as a part of panel of five SNPs (single SNPs in genes *ABCB1*, 5,10-Methylenetetrahydrofolate reductase (*MTHFR*, 607093), Glutathione *S*-transferase, pi (*GSTP1*, 134660), and two SNPs in Xeroderma pigmentosum, complementation group C (*XPC*, 613208) modifying progression-free survival of 124 locally advanced EC (62 with ESCC and 62 with EAC) patients who received neoadjuvant chemoradiation based on cisplatin, docetaxel, and 5-fluorouracil [18]. Although Okuno et al. found no association of rs1045642 SNP with response rate or survival of ESCC patients [19], Narumiya et al. have analyzed impact of *ABCB1* rs1045642 SNP on outcome of EC patients (116 with ESCC and 146 with EAC) after neoadjuvant treatment with cisplatin- and 5-fluorouracil-based chemoradiation and observed that CC genotype was associated with lymph node and distant metastases and predicted shorter overall survival [14]. These differences could be due to different frequency of rs1045642 SNP in studied populations, Caucasian (Narumiya et al.) [14] versus Japanese (Okuno et al.) [19]. Additionally, the analysis of 116 ESCC patients in the former study demonstrated no significant role for *ABCB1* genotype in terms of survival of patients, pointing to a possible difference between histological types of EC [14]. Interestingly, *ABCB1* rs1045642 SNP did not associate with lansoprazole plasma concentrations in 51 Japanese patients with EC of unspecified histology. Lansoprazole pharmacokinetics was affected by SNP in Cytochrome P450 2C19 (*CYP2C19*, 124020), but not by genetic variability in *ABCB1* gene [20]. However, a recent systematic review and meta-analysis of potential genetic biomarkers in EAC and ESCC concluded that rs1045642 in *ABCB1* may be considered a putative biomarker of survival or recurrence that merits further investigation [21].

The etiology of EC is not yet fully understood. According to the most recent studies [47,48], the pathogenesis of ESCC includes endogenous factors such as immune system as well as genetic variation. From this point of view, frequently studied genes in ESCC *TAP1* (ABCB2, 170260) and *TAP2* (ABCB3, 170261) encoding transporters associated with antigen processing (antigen processing machinery, APM) might modify prognosis of ESCC, especially in the view of high prevalence of human papillomavirus (HPV) in ESCC [49]. Cao et al. reported an association of SNPs in large multifunctional protease (LMP) genes *LMP2* (177045) and *LMP7* (177046) and APM genes *TAP1* and *TAP2* with HPV infection and ESCC. Patients carrying rs1800454 (Val379Ile) SNP in *ABCB2* had higher risk of developing ESCC, which was further strengthened in homozygous *LMP7*-reference/*TAP2*-variant haplotype in patients positive for HPV infection [24]. Another *TAP1* SNP, rs1135216 (Asp637Gly) also associated with the risk of ESCC in a recent study. Study addressing role of rs1135216 and rs1057141 (Ile333Val) SNPs in 361 ESCC patients and 66 healthy controls reported that patients with alternative allele G in rs1135216 had significantly higher risk of ESCC, but lower risk of developing large (T3 or T4) tumors [22]. Association of germline genetic variability in *TAP1* with etiology and progression of ESCC was further corroborated by study observing a significantly higher frequency of rs1135216 AG genotype in 200 ESCC patients compared to 100 healthy controls. Moreover, carriage of AG genotype also associated with the presence of lymph node and distant metastasis [23].

Recently, high-density SNP arrays were employed for genetic studies of human cancers and identified several new potential factors in cancer therapy. In one such study, SNPs in 225 genes have been evaluated in a cohort of 116 patients (56 EAC and 60 ESCC) treated in neoadjuvant setting with platinum-based regimens and radiotherapy. SNPs rs17222723 in *ABCC2* (MRP2, 601107) and rs2277624 in *ABCC3* (MRP3, 604323) along with SNPs in Cytochrome P-450 2A6 (*CYP2A6*, 122720), Peroxisome proliferator-activated receptor-gamma (*PPARG*, 601487), and Solute carrier transporter 7A8 (*SLC7A8*, 604235) had predictive power to distinguish between poor and good responders [25].

Large scale meta-analysis of genome wide association studies (GWAS) performed by high-density SNP arrays of DNA samples from patients with EAC or pre-malignant BE and healthy controls discovered new risk loci. The rs17451754 near *CFTR* (ABCC7, 602421) gene was predictive for both adenocarcinoma and BE risk and rs9823696 located near 5-Hydroxytryptamine receptor 3C (*HTR3C*, 610121) and *ABCC5* (MRP5, 605251) specifically predicted adenocarcinoma risk [50].

Recently, out of 57,774 individual CNVs identified in a study analyzing 128 discordant sibling pairs, several ABC transporter genes (*ABCA1*, 600046; *ABCA8*, 612505; *ABCC4*, 605250; *CFTR*) have been reported to carry CNVs. Amplification of *ABCC4* was also detected in independent cohort (*n* = 1048) of Han Chinese subjects and significantly associated with ESCC risk (odds ratio: 3.36, 95% confidence interval: 1.65–7.93, *p* = 0.001). Moreover, high ABCC4 protein expression levels correlated with increased CNVs and predicted a poor overall survival of ESCC patients (*p* = 0.018) [26] similar to a previous study reporting the correlation of *ABCC4* gene amplifications with poor survival of EAC patients [51]. Thus, ABCC4 which is involved in tumorigenesis by regulating prostaglandin E2-related pathways (e.g., Wnt) and immune responses may help predict prognosis of EC and provide a potential therapeutic target.

Thus far, rather fragmentary genetic studies suggest that EC etiology and clinical outcome need to be studied in the population and histology specific context. EC pharmacogenomics cannot be fully understood without more complex and global approach.

#### 2.1.2. Somatic Aberrations

Data on somatic aberrations in 2885 tumor samples (out of 12,247 totally screened) with primary site esophagus and carcinoma histology available in COSMIC database [27] show that majority of gene mutations are missense substitutions (76%) followed by nonsense (39%) and synonymous (33%) substitutions. Structural variations as frameshift and inframe insertions or deletions and complex mutations range between 1% and 23%. Basically, all ABC transporter genes are mutated in EC tumors (Table S1), but functional and clinical relevance of these aberrations has not yet been addressed.

#### 2.1.3. Structural Variations in ABC Transporters Specifically Studied in Esophageal Cancer

All multidrug resistant subclones of ESCC cell line SH-1 contain increased CNV in chromosomal location 16p including *ABCC1* (MRP1, 158343) gene and the most resistant subclones (SH-1-V4 and SH-1-V8) also harbor amplification of 7q11.1-22 region where *ABCB1* resides [52]. The latter has previously been shown to overexpress ABCB1 transcript [53] implicating the *ABCB1* amplification and overexpression in resistance development. In total, increased CNV of *ABCB1* in roughly 40% of Barrett’s adenocarcinoma tumor tissues (*n =* 18) [54] and recurrent amplification of *ABCB1* in adenocarcinomas of the gastroesophageal junction (*n =* 14) has been reported [55] documenting that at least *ABCB1* CNV is quite frequent in gastrointestinal malignancies and thus relevant for EC in general.

Structural variations in other ABC transporters have been described rather rarely in EC. Frequent amplification of chromosome 3q region including *ABCC5* gene in study using 33 archival ESCC samples [56] may serve as example.

As apparent from studies reviewed, except for publicly available databases containing repository of somatic aberrations identified by whole-genome or whole-exome sequencing in EC tumors from patients or quite a small number of cell line models, there are scarce data on relevance of mutational spectra of ABC transporters for clinical outcome in human EC. The utility of whole somatic genome screening for EC may be demonstrated by a recent study reporting somatically acquired mutations in Tumor protein p53 (*TP53*, 191170), Semaphorin 5A (*SEMA5A*, 609297) and *ABCB1* genes in both parental EAC tumor and derived cell line model MFD-1 [57]. Such approach may well be used not only for cell line authentication, but also for characterization of functionality of genetic aberrations with subsequent implications for experimental therapeutics and targeted therapy design.

### 2.2. Epigenetic Dysregulation

DNA methylation, acetylation/deacetylation of histone proteins and dysregulation of microRNA expression present the most frequently studied epigenetic factors.

#### 2.2.1. DNA Methylation

DNA methylation profiling studies have shown that, similar to other cancer types, the EC genome contains focal areas of hypermethylation and widespread areas of hypomethylation, compared with nonmalignant esophageal mucosa. These epigenomic aberrations contribute to the pathogenesis of EC, through different mechanisms. Most studies focused on the promoter hypermethylation of tumor suppressor genes and their effect in early steps of ESCC carcinogenesis and their importance for prognosis of ESCC. Hypermethylation was observed mainly for *CDKN2A* (600160) and other tumor suppressor genes (e.g., *APC*, 611731; *RPRM*, 612171; *IGF2*, 147470; *RASSF1A*, 605082; *SFRP1/2/4/5*, 604156/604157/606570/604158; *EYA4*, 603550; *DAPK*, 600831; *RUNX3*, 600210; and *UCHL1*, 191342), genes related to cell adhesion (*CDH1*, 192090; *CDH13*, 601364; *CLDN3*, 602910; *CLDN4*, 602909; *DCC*, 120470; *LRP1B*, 608766; *PCDH10*, 608286; *PCDH17*, 611760; and *TSLC1*, 605686), DNA repair genes (*MGMT*, 156569; *MLH1*, 120436; and *FHIT*, 601153) and growth factor response-related gene *RARB* (180220) [58,59,60]. Promoter methylation of tumor suppressor genes *CDKN2A*, *FHIT*, *MGMT* [58,61], *HIN1* (606500) [62], and *TFF1* (113710) [63] occurs in early stages of ESCC and might therefore serve as biomarkers for early diagnosis.

Furthermore, a specific *CDKN2A* promoter methylation was detected in both tumor tissue and serum fluid pairs in 18% of ESCC patients [64]. Subsequently, a panel of methylated tumor suppressor genes including *RARB*, *DAPK*, *CDH11* (600023), *CDKN2A*, and *RASSF1A* was detected with high specificity in serum DNA of ESCC patients [65]. Thus, an analysis of promoter methylation of such panel of tumor suppressor genes may be relevant for early detection of ESCC [66] and represent first potential clinical implication of epigenetic changes in ESCC.

Hypermethylation of tumor suppressor genes has also been described as a biomarker of therapy outcome, e.g., a hypermethylation of the *APC* promoter in ESCC patients was significantly associated with reduced survival times [67] and promoter hypermethylation of *CDH1* and *ITGA4* (192975) with ESCC recurrence [68,69].

On the other hand, global hypomethylation is much less studied. Until now, only a few genes have been found hypomethylated in ESCC, including, *SLC22A17* (611461) [70], *GADD45α* (126335) [71] and Long interspersed nuclear elements (*LINE-1* or LRE1, 151626) [72,73]. *LINE-1* belongs to group of transposable elements contributing to gene diversity and its hypomethylation was associated with increased chromosomal instability, *TP53* mutation rate, lymph node metastasis [74], as well as shorter survival times of ESCC patients [72].

Very little is known about the methylation status of ABC transporter genes in EC. The only study published so far revealed an association of an elevated expression of ABCB1 gene with hypomethylation of *LINE-1*, indicating poor prognosis of ESCC patients [33]. ABCB1 transcript levels have been investigated in 310 patients with ESCC after surgical resection and negatively correlated with *LINE-1* methylation and independently predicted poor overall survival in multivariate Cox regression analysis. Additionally, *LINE-1* hypomethylation status alone also associated with shorter overall survival, higher stage, grade and older age of patients [33]. This relationship suggests that aberrant expression of ABCB1 might be epigenetically regulated in ESCC.

Our understanding of the pathogenesis of EC has increased markedly, but importance of perturbations of the DNA methylome of ABC transporters associated with multidrug resistance phenomenon is completely unknown. Characterization of the DNA methylation of ABCs and its role in EC carcinogenesis will help to better understand the etiology and help in developing more efficient therapies.

#### 2.2.2. Histone Modifications

Epigenetic regulation of gene expression by histone modifications is one of the mechanisms of human carcinogenesis [75,76]. Histone modifications include acetylation, methylation, phosphorylation and ubiquitination usually occurring at the terminal tails of histone proteins protruding from nucleosomes [77]. Reversible histone methylation and acetylation modifications are catalyzed by three types of enzymes including histone acetyltransferases (HATs), histone methyltransferases (HMTs) and histone deacetylases (HDACs) [76,78]. In general, acetylation of histone tail destabilizes the chromatin structure. Hypoacetylated histones are involved in gene silencing, while hyperacetylated ones play a role in gene activation [78,79].

Investigation of epigenetic histone modifications in EC was mainly focused on acetylations/deacetylations and methylation of histone proteins H3 and H4 and altered expression of histone-modifying enzymes HDACs. Histone H4 hyperacetylation was found in early stages of ESCC and changed into a hypoacetylated state with cancer progression [80]. Acetylation levels of histone H4 inversely correlated with better prognosis [81]. More specifically, correlations of expression of histone modification markers: acetylated histone 3 lysine 18 (H3K18ac), dimethylated histone 4 lysine 3 (H4K3me2), trimethylated histone 3 lysine 27 (H3K27me3) and dimethylated histone 4 arginine 3 (H4R3me2) with prognosis of ESCC were reported [82,83] and H3K27me3 appeared to be an independent biomarker of improved prognosis in early stages ESCC [82]. Later, the results of analyses of specific histone markers by immunohistochemical staining have been reviewed [60,66].

Among the histone-modifying enzymes, the down-regulation of HDAC1 (601241) in ESCC in comparison to normal epithelium was described, but the low expression of HDAC1 in ESCC tissues significantly increased as tumor cells invaded into the deeper layers of the esophageal wall [80] and seemed to be associated with worse prognosis of ESCC. Finally, significant associations between germline genetic variation of the total histone and acetylation pathway (89 genes) and ESCC risk have recently been described by [76] suggesting that the role of histone modifications in ESCC prognosis and therapy outcome needs to be further investigated in more detail.

#### 2.2.3. MicroRNAs

MicroRNAs (miRNAs) are short non-coding single-strand RNAs that post-transcriptionally regulate target mRNA through the binding to complementary sites in 3′-UTR region. miRNAs are involved in numerous cellular processes including development, proliferation, differentiation or apoptosis, and are dysregulated in a variety of cancers including EC [28,84,85,86]. Published data support the hypothesis that miRNAs are involved in tumor formation, proliferation, and responsiveness to treatment, and therefore influencing survival of patients. In recent years, a series of review articles have been published summarizing data on miRNAs with possible oncogenic or tumor-suppressor function in EC or miRNAs affecting sensitivity to therapy of this cancer [87,88,89,90,91]. Thus, available studies on the role of miRNAs in chemotherapy resistance of EC in connection with ABC transporters are summarized below.

Several in vitro studies showed an involvement of miRNAs in resistance to chemotherapy in EC [92,93,94,95,96,97]. In a pioneering study, Zhang et al. hypothesized that miR-27a could be related to chemoresistance in human ESCC in vitro models, ECA-109 and TE-13 due to increased chemosensitivity to vincristine and adriamycin, reduced expression of ABCB1 transcript and protein, and deregulated expression of apoptotic genes Bcl-2 (*BCL2*, 151430) and Bax (*BAX*, 600040) observed after the treatment of cells by the miR-27a antagomir [98]. With the help of cisplatin-resistant cell line model of ESCC, (KYSE cell lines) Imanaka et al. showed that miR-141 is involved in resistance to cisplatin-induced apoptosis through negative regulation of Yes-associated protein 1 (*YAP1*, 606608), pro-apoptotic transcription factor [92]. Using 5-fluorouracil-resistant cell models of EAC (established from OE19, OE33, PT1590 and LN1590), miR-221 was identified to be involved in resistance to 5-fluorouracil by direct targeting of Dickkopf (*DKK2*, 605415) which is known inhibitor of Wnt/b-catenin signaling pathway and modulation of miR-221 expression substantially dysregulated, among others, gene expression of ABCG2 (BCRP, 603756) [97]. miR-214 enhanced sensitivity of ESCC model TE7 to cisplatin by direct targeting survivin (*BIRC5*, 603352) and RNA-binding protein 1 (*CELF1*, 601074) [99].

Cisplatin and 5-fluorouracil resistant cell line models of ESCC and EAC have been established [93] and profiled using microarrays, which allow measurement of hundreds of miRNAs or whole miRNome (all known miRNAs of studied species) at the same time. After the evaluation by qPCR, eleven miRNAs have been found dysregulated in cisplatin-resistant subclone of esophageal adenocarcinoma OE19 cells and 19 miRNAs have been dysregulated in 5-fluorouracil-resistant subclone. Similarly, analysis of cisplatin- and 5-fluorouracil-resistant subclones of ESCC KYSE410 cell line has shown dysregulation of one and six miRNAs, respectively. None of miRNAs has been deregulated in the same way in all studied cell subclones which indicates different function of individual miRNAs in both EAC and ESCC tumor types as well as in the resistance [94]. A downregulation of let-7c was one of 15 miRNAs deregulations in two cisplatin-resistant cell line models (TE8 and TE10) of ESCC [96]. Moreover, transfection of let-7c significantly increased sensitivity of these cells to cisplatin-induced apoptosis, most probably through repression of the IL-6/STAT3 pathway, a pro-survival pathway activated by genotoxic agents as, e.g., cisplatin [96]. The fact that the let-7 family of miRNAs was similarly deregulated in cisplatin-resistant cell models used in studies reported by Sugimura et al. and Hummel et al. indicates a large variability of results obtained by these two epigenomic studies [94,96]. Interestingly, overexpression of let-7g and let-7i significantly inhibited proliferation of ECA-109 and TE-10 cell line models of ESCC and promoted cisplatin-induced apoptosis and ABCC10 (MRP7, 612509) has recently been identified as a functional and direct target gene for let-7g and let-7i [100].

Other studies investigated involvement of miRNAs in chemotherapy resistance also in clinical tumor samples from EC patients [28,29,95,96,98,101,102]. miR-200c and the above mentioned let-7c expression were associated with the response of ESCC patients to neoadjuvant treatment based on cisplatin, Adriamycin, and 5-fluorouracil [95,96]. High intratumoral levels of miR-296 significantly associated with shorter disease-free survival of ESCC patients [28], and inhibition of miR-296 with antagomir in ECA-109 cell line caused change in expression of cell cycle-related genes Cyclin D1 (*CCND1*, 168461) and p27 (*CDKN1B*, 600778). At the same time, decreased expression of ABCB1 drug efflux transporter and main regulator of apoptosis BCL2 has been observed. The inhibition also led to increased sensitivity of model cell line to substrates of ABCB1, anticancer drugs vincristine and doxorubicin. On the other hand, cells have also been sensitized to 5-fluorouracil and cisplatin which are not translocated by ABCB1, indicating that miR-296 probably plays complex role in ESCC proliferation and chemotherapy resistance [28]. Furthermore, miR-483 and miR-214 overexpression in ESCC patients leads to chemotherapy resistance, potentially mediated by ABCB1 regulation [29]. Odenthal et al. [101] followed differences in pre- and post-treatment intratumoral levels of miRNAs between EC patients with good and poor response to neoadjuvant therapy based on cisplatin and 5-fluorouracil. High pre-treatment levels of miR-192 and miR-194 were associated with good response to neoadjuvant therapy but only in ESCC patients in contrary to patients with EAC [101]. Higher intratumoral levels of miR-141, previously connected with resistance to cisplatin-induced apoptosis [92], were associated with more advanced stage and higher grade in ESCC patients. miR-141 was also upregulated in ESCC cell line EC-9706R resistant to oxaliplatin and 5-fluorouracil (established from EC-9706) in vitro, and its inhibition sensitized EC-9706R cells to both drugs through regulating its downstream target Phosphatase and tensin homolog (*PTEN*, 601728) in vivo [102].

Many studies focused on blood-based biomarkers as a minimally invasive method for the early detection of the primary tumor, minimal residual disease, relapse or detection of drug resistance [103]. Circulating tumor cells, cell free nucleic acids and proteins were studied in whole blood, serum or in plasma of EC patients as potential more specific and sensitive biomarkers compared to conventional serum tumor markers like Carcinoembryonic antigen (CEA), Squamous cell carcinoma antigen (SCC) or Cytokeratin-19 fragment (CYFRA 21-1, CY21-1). miRNAs are the most intensively investigated circulating biomarkers because of the relative resistance to high ribonuclease activity present in serum and plasma that makes detection of longer RNA fragments difficult [104]. Most effort has been devoted to search for biomarkers allowing a faster diagnosis of EC and the monitoring of tumor dynamics [105,106,107,108,109,110,111]. Tanaka et al. followed the results of the study reported by Hamano et al. [95] which was based on tumor samples and evaluated levels of miR-21, miR-145, miR-200c and let-7c in serum of 64 patients with ESCC. An association with the response of ESCC patients to neoadjuvant chemotherapy based on cisplatin, 5-fluorouracil and Adriamycin or cisplatin, 5-fluorouracil and docetaxel was analyzed. High levels of miR-200c (which belongs to the same miRNA-200 family as the above mentioned miR-141) was significantly associated with shorter progression-free survival of patients and inferior response to the neoadjuvant therapy [112].

Several validated miRNAs target major drug resistance-related ABC transporters (ABCB1, ABCC1, and ABCG2) in different types of carcinomas [113,114,115]. Two miRNAs downregulating ABCB1, miR-508-5p and miR-129-5p, are expressed in gastric cancer cell lines [116,117] and may be relevant for EC. Similarly, miR-595 sensitizes ovarian cancer cells to cisplatin by directly targeting ABCB1 [118]. ABCB1 and ABCC1 may be downregulated by miR-145 [119,120], one of the most upregulated miRNAs in EAC [85]. Interestingly, two out of six miRNAs most downregulated in EC-9706R cells resistant to oxaliplatin and 5-fluorouracil, miR-222 and miR-345 [102], are validated modulators of ABCG2 and ABCC1 expression, respectively [121,122].

Thus, reported data indicate that several miRNAs targeting ABC transporters may be involved in chemotherapy resistance of EC and their role should be further studied.

### 2.3. Phenotype Changes

As already mentioned above, the expression of ABC transporters is usually increased in patients with chemoresistant cancer and may serve as predictive biomarker for the selection of the optimal therapeutic regimen. The resistance of tumor cells is either inherent, i.e., present in chemotherapy-naïve cells or acquired during repeated dosing of chemotherapy. Both types may have genetic and/or epigenetic background, but, at present, the analysis of either transcript or protein expression (phenotype) in tumor cells is the preferred way of detection.

#### 2.3.1. Transcript Expression

Gene expression of ABC transporters is significantly associated with response to therapy of cancer patients and, consequently may serve as an independent prognostic biomarker.

ABCB1 transcript was firstly monitored using northern blotting in 169 human tumor specimens of different histology types. This panel of tumors included five esophagus specimens (normal and malignant tissue pairs), but no ABCB1 signal in these samples was detected [123]. Later, a study based on reverse transcription polymerase chain reaction (RT-PCR) approach reported low levels of ABCB1 gene expression in two ESCC resection specimens [124]. Zhang et al. studied tumor samples from 46 patients with ESCC and, using RT-PCR, observed ABCB1 transcript expression in 37% of samples, with no correlation with grade or stage of tumors [29,30]. ABCC1 gene expression was found higher in ESCC tumors (*n =* 25) compared to normal tissues by quantitative RNase protection assay and this result was confirmed on the protein level [125]. Thus, questions about expression of two major ABC transporters have been solved and search for clinical relevance followed.

Di Nicolantonio et al. [126] evaluated gene expression of 16 genes, including ABCB1, ABCC1, ABCC2 and ABCG2, in 34 paired EAC biopsies. Biopsies were obtained before and after administration of two cycles of epirubicin-, cisplatin- and 5-fluorouracil-based neoadjuvant chemotherapy in 18 EAC patients. An increase in the expression of ABCB1 transcript was observed in EAC tumor samples after chemotherapy compared with the paired biopsies before (*p <* 0.001) suggesting induction of gene expression of ABCB1 (and protein expression analyzed by immunohistochemistry) by administration of drugs [126]. Simultaneously, high ABCC1 transcript levels were significantly associated with longer overall survival in 38 patients with locally advanced (T3N1) EAC and with the response to neoadjuvant chemotherapy based on cisplatin and 5-fluorouracil [35]. Gene expression of ABCB1 and ABCC1 was later measured in 31 T3N1 EAC preoperative biopsies and compared with surgical resection specimens after therapy and significant reduction in transcript levels of ABCC1 was observed in post-therapy specimen [31]. In the follow up study, same authors also analyzed gene and protein expression of ABCB1 and ABCC1 in 40 patients with T3N1 EACs treated with cisplatin and 5-fluorouracil. High protein level of ABCC1, but not of ABCB1 was associated with poor response to neoadjuvant chemotherapy [32]. However, despite a previous report of correlation between ABCC1 transcript and protein levels in 14 patients with ESCC [127], no such correlation for ABCC1 or ABCB1 has been observed by Langer et al. [32,35]. Thus, ABCB1 and ABCC1 gene expression levels seem to predict response and survival of EAC patients and should be further studied as putative prognostic and predictive biomarkers in EAC.

Only limited data are available about the role of other ABC transporters in EC. Downregulation of ABCB2 and ABCB3 transcript and protein levels was observed in 11 of 16 biopsies from EAC patients [128]. Transcript and protein levels of ABCC2 seemed to be induced by neoadjuvant treatment with 5-fluorouracil, doxorubicin and cisplatin in ESCC patients and correlated with poor response of patients to the therapy. Thus, the authors hypothesized that ABCC2 may be responsible for acquired chemoresistance, which was further supported by the observation of reduced chemoresistance to cisplatin by siRNA directed ABCC2 knockdown in TE14 cell line in vitro [36]. This result corroborates the previous study in which higher ABCC2 expression in 36 EC (32 EAC and 4 ESCC) samples from patients treated in neoadjuvant setting with oxaliplatin and 5-fluorouracil associated with decreased progression-free survival of patients [129]. Increased transcript levels of ABCC3 in BE and EAC (*n =* 37) compared to normal squamous epithelium were found. ABCC3 protein expression was also increased in BE (not detected in normal mucosa) and decreased with progression to EAC [130].

ESCC patients with higher expression of ABCG2 transcript (levels higher or lower than tumor/normal tissue ratio 1) or positive protein staining had significantly shorter overall survival, but ABCG2 expression has not influenced the effect of cisplatin-based neoadjuvant treatment [37]. In another study, microarray chip profiling was performed on eight pairs of ESCC tissues and matched esophageal mucosal epithelial tissues. Gene expression of ABCA8 (612505) was downregulated in tumor tissues and using pathway analysis, *ABCA8* was identified among seven genes playing a key role in the signal transduction networks potentially associated with ESCC [131].

An in vivo study on ESCC cell lines ECA-109 and ECA-9706 has been published recently in a mouse model. The expression of ABCB1, ABCC1, and ABCG2 was increased in tumors from human xenografts with properties of sphere formation stem-like cells compared with parental cell forming tumors. The mechanistic study revealed that Signal transducer and activator of transcription 3 (*STAT3*, 102582) regulated proliferation of sphere forming cells and increased colony formation of spheroid cells by *trans*-activating miR-181b [132]. Whereas cancer stem cells can be resistant to chemotherapy, this study supports a previous result where the ABCB1 (together with Topoisomerase II, *TOP2A*, 126430) levels were decreased in xenografts of ESCC cells lines EH-1 and EH-6 sensitive to doxorubicin [133]. In another study, ex vivo experiment in cell lines established from 54 patients with unspecified EC histology showed different modulation of ABCB1 expression via Hypoxia-inducible factor 1α (*HIF1A*, 603348). ABCB1 transcript expression increased after irradiation with 28 Gy, but on the contrary, ABCB1 expression decreased after 7 Gy irradiation [34]. Several studies were performed in vitro on various EAC and ESCC cell lines. The most widely studied ABC transporter is ABCB1. ABCB1 function has either been studied alone [133,134,135], in combination with ABCC1 [136,137] or together with both ABCC1 and ABCG2 [138,139]. ABCE1 (*RNS4I*, 601213), ABCC2 and ABCC5 belong to other ABCs studied in vitro [140,141,142]. Minegaki et al. studied expression of genes affecting the sensitivity of EAC and ESCC cell lines to 5-fluorouracil and cisplatin. This study included ABCB1, ABCC1-6, and ABCG2. Resistance to 5-fluorouracil was associated with transcript levels of ABCB1 and ABCC2. On the other hand, cisplatin resistance correlated with ABCC2 level [143]. Studies of ABC in EC cell line models are summarized in Table S2.

There is plethora of studies investigating drug resistance and prognosis of EC connected with a single ABC transporter transcript level, but except ABCB1 and ABCC1 no putative biomarkers have yet been identified. Nevertheless, the next-generation sequencing era is making the space open for a more complex profiling approach, e.g., clustering of all resistance-connected transporters or transporters with different biochemical functions [144] for a more precise estimation of prognosis and prediction of therapy outcomes.

#### 2.3.2. Protein Expression

Proteins are responsible for the enzymatic activity or other molecular functions, including structural support, transporting, signal transduction, immune response and other, and therefore protein expression levels are important determinants of response of cells and tissues to therapy. Thus, the determination of protein levels is still preferred in clinical setting and represents a major prognostic or predictive tool. Immunological methods such as immunohistochemistry (IHC), immunoblotting or (enzyme-linked immunosorbent assay) ELISA-based ones are routine for each pathology laboratory.

Similar to associations between ABCs transcript levels with EC prognosis and therapy outcome reported to date, observations on the protein level are quite scarce.

Although initially Robey-Cafferty et al. reported that ABCB1 protein expression in EAC tumors after neoadjuvant chemotherapy may be predictive for response of patients and thus tumor sensitivity to chemotherapy [145], the follow up study concluded that chemotherapy-induced changes in morphology seriously complicate analysis in post-chemotherapy specimens. Similarly, further studies suggested that, unlike in other cancers (e.g., breast, colorectal, gastric and ovarian carcinomas), protein expression of ABCB1 has “no value in predicting the responsiveness of the tumor to chemotherapy or radiotherapy in squamous cell cancers” [146] based on the own data and previous data of [147]. However, in line with the study size concerns (Darnton et al. [147] comment to Sur et al. [146]), more recent studies have shown a prognostic and predictive potential of ABCB1. First, high ABCB1 protein expression has been identified as an independent predictor of early recurrence and death for EAC and ESCC patients (*n =* 118) treated with chemoradiotherapy based on 5-fluorouracil and cisplatin [38]. Subsequently, ABCB1 protein overexpression has been observed in half of EAC patients (*n* = 16) whose biopsies were taken before and after treatment with combination of epirubicin, cisplatin and 5-fluorouracil [126].

Despite the potential prognostic role, experimental evidence indicates that silencing of ABCB1 overexpression by siRNA restores sensitivity in taxane-resistance of ESCC cell line models in vitro [148]. Previous studies have already suggested important role for ABCB1 protein overexpression in paclitaxel- and cisplatin-resistance in radio-resistant EC-9706 ESCC model in vitro [149]. Interestingly, the resistance to taxane could be blocked by ABCB1 inhibitor verapamil, but resistance to cisplatin remained unaffected [149], suggesting involvement of additional mechanism(s).

ABCC1 protein has been detected in ESCC tumors (*n* = 12) using IHC for the first time by [125]. A subsequent study observed ABCC1 protein expression in more than 60% of patients with ESCC (*n =* 86) and this rate exceeded that of stomach and colorectal adenocarcinomas [150]. In the same year Nooter et al. reported correlation between ABCC1 protein and transcript expression levels and the increased intratumoral expression after neoadjuvant chemotherapy of ESCC patients with cisplatin and etoposide compared with diagnostic pre-treatment biopsies [127]. High ABCC1 protein expression in pre-treatment tumor biopsies significantly correlated with poor response of EAC patients (*n =* 40) to 5-fluorouracil- and cisplatin-based neoadjuvant therapy, while ABCB1 expression had no predictive value in the later study [32]. However, largest study to date failed to observe any prognostic association for immunohistochemically detected ABCC1 protein expression in tumors from ESCC patients (*n =* 829) [41]. Most recently, correlation of ABCC1 and ABCB1 protein expression with that of Caveolin-1 (*CAV1*, 601047) in tumor tissues from ESCC patients (*n =* 84) has been observed and CAV1 expression knockdown also decreased transcript levels of both transporters in ECA-109 cell line in vitro [151], suggesting a potential interplay between membrane proteins with different functions and their putative use as therapeutic targets. In summary, although predictive value of ABCC1 protein expression has been suggested, studies aiming at its prognostic role failed [40,41] and, therefore, other members of the C family and perhaps combinations of more transporters should be followed in the future.

In analogy to ABCB1 and ABCC1, an association of ABCG2-positivity (any protein expression) in tumors from ESCC patients (*n =* 100) with the poor survival (*p* = 0.009) has been demonstrated [37]. Subsequent studies confirmed both ABCG2 protein overexpression in majority (75%) of EC (36 EAC and 4 ESCC) tumor tissues [152] and poor prognosis (short overall survival) of ESCC patients (*n* = 110) with high ABCG2 protein, especially early stage disease [45]. ABCG2 protein expression in ESCC tumor tissues (*n* = 66) and the correlation with disease stage, grade and metastasis was confirmed by another study [153]. A mechanistic study indicated that enhanced expression of ABCG2 detected in EC tumors may be attributed to the interaction of specific ligands, e.g., components of cigarette smoke, with Aryl hydrocarbon receptor (*AHR*, 600253), Transcription factor Sp1 (*SP1*, 189906), or Nuclear erythroid 2-like 2 (*NFE2L2* alias Nrf2 600492) within the *ABCG2* promoter sequence [154]. Additionally, another study reported that AHR activation establishes chemoresistance through upregulation of ABCG2 and AHR antagonists such as kaempferol can reverse this effect [155], bringing together the cancer predisposition and therapeutic roles of ABCG2. Lastly, ABCG2 protein overexpression accompanied by increased drug efflux rate resulted in resistance of ECA-109 cell line model to doxorubicin in vitro [156] providing additional rationale for future individualization of EC therapy. This resistance could be reversed, through downregulation of ABCG2, by administration of epigallocatechin-3-gallate in vitro [157].

ABC transporters other than ABCB1, ABCC1 and ABCG2 were much less studied in EC. A gradual decrease of ABCC3 protein expression in the spectrum ranging from non-dysplastic BE to dysplastic BE and EAC, observed by Dvorak et al. [130], suggested that transport of bile acids is linked to esophageal carcinogenesis [130]. However, this observation was not further replicated or investigated. A large IHC study of ABCB1 and ABCC2 protein expression in ESCC tumors (*n* = 582) has shown a significant increase in ABCC2 protein level with pathological grade and positive correlation between ABCB1 and ABCC2 levels suggesting an indirect prognostic potential of both transporters for ESCC patients [42]. A more recent study reported prognostic role of ABCC2 in ESCC patients (*n* = 81) documented by the fact that patients with ABCC2 expressing tumors had poorer overall survival than patients without expression. Simultaneously, correlation of ABCC2 expression with resistance to cisplatin in ESCC cell model in vitro was observed [36].

Antigen presentation machinery, an important component of immune response to cancer cell (e.g., recruiting cytotoxic T lymphocytes), depends on proper function of ABCB2 and ABCB3 transporters. ABCB2 and ABCB3 protein expression was lost or downregulated in roughly 30% of immunohistochemically evaluated ESCC tumors (*n* = 143) and the expression of ABCB2 significantly correlated with tumor grade and presence of metastasis in regional lymph nodes [39]. In another study, ABCB3 transcript and protein expression has been found downregulated in 70% of EAC tumors from patients and could be restored by Interferon-gamma (*IFNG*, 147570) in OE19 cells in vitro [128] suggesting a promising opportunity to combine immunotherapy and cytotoxic therapy in EAC and potentially other cancers.

ABCE1 is not typical efflux transporter, but rather a key promoter of eukaryotic ribosomal recycling because of the ability to split the 80S ribosomes into 60S and 40S subunits [158]. ABCE1 overexpression in tumors compared with non-neoplastic mucosa of ESCC patients (*n* = 112) was accompanied by reduced expression of its target Ribonuclease L (*RNASEL*, 180435) and significantly correlated with grade and stage of ESCC tumors. Moreover, downregulation of ABCE1 expression by siRNA resulted in reduced proliferation, migration and enhanced apoptosis rate in ECA-109 cells in vitro [44] suggesting that, together with some other ABCs mentioned above, this molecule may represent a potential therapeutic target in EC.

Last, but not least, an increased protein expression may induce production of auto-antibodies suggesting an interesting opportunity for personalized medicine. Indeed, a significantly higher titer of circulating IgA autoantibodies to ABCC3 has been found by ELISA in ESCC patients (*n* = 114) compared to healthy control subjects (*n* = 226), suggesting the diagnostic and predictive potential of this approach [43]. Considering this exciting observation and the fact that determination of antibody titer can be performed simply from blood sample, such approach represents a more convenient and significantly cheaper technique for EC screening and detection of tumor response compared with repeated upper gastrointestinal endoscopy or imaging.

Available studies in the literature suggest that expression of several ABCs proteins correlates with prognosis (ABCB1, ABCC2, and ABCG2) or response to therapy (ABCC1) of EC patients (Table 2). However, the lack of replication studies and, similar to transcriptomic data, poor awareness about complex interplay between both efflux and uptake transporters and eventually other pathways connected with drug resistance still precludes any meaningful generalization and clinical use.

### 2.4. Signaling Pathways

It is evident that ABC transporters-mediated chemoresistance is very complex phenomenon that seems to be regulated or co-regulated by various signaling pathways and mediated by several proteins with either oncogenic or tumor suppressor functions.

For example, silencing of Y-box-binding protein 1 (*NSEP1*, 154030) inhibits cell proliferation and invasion by downregulating genes from E2F, PI3K/Akt/mTOR, and MAPK pathways, enhances chemosensitivity to cisplatin, and reduces ABCB1 protein expression in ECA-109 and TE-1 ESCC cell models in vitro [159]. Another signaling pathway involved in regulation of ABC expression is Wnt/β-catenin pathway. Ouabain, a cardiac glycoside, downregulated ABCB1 expression by inhibiting the translocation of Wnt/β-catenin into the nucleus and thereby reversed the multidrug resistance of ECA-109 cell subline resistant to cisplatin [160]. In the most recent study, knockdown of Frizzled-7 (*FZD7*, 603410) promoted chemosensitivity to cisplatin in ESCC cells ECA-109, TE-10, and TE-11 and suppressed ABCB1 expression by inhibiting Wnt/β-catenin signaling [161].

Furthermore, Hedgehog pathway regulates tissue growth and differentiation during embryogenesis. Its relation to tumor growth has already been proven and specific inhibitors (e.g., vismodegib) are in clinical use. Sims-Mourtada et al. investigated potential relation between sonic hedgehog pathway and ABC transporters and have shown that activation of this pathway induces chemoresistance and the knockdown reduces expression of ABCB1 and ABCG2 and partly restores chemosensitivity [162].

Similar to Hedgehog, the Caudal-type homeobox transcription factor (*CDX2*, 600297) also has an important role in embryonal development and differentiation (especially of intestine) and was implicated in human colon cancer. Takakura et al. demonstrated a direct regulation of ABCB1 gene expression by CDX2 in various colorectal cancer cell lines and human tumor tissues. The observation of connection between enhanced expression of CDX2 in HT-29 cells in vitro with resistance to vincristine and paclitaxel and its reversal by ABCB1 inhibitor, verapamil further substantiated the role CDX2 in ABCB1-mediated drug resistance [163].

Studies addressing the embryonic stem cell network have recently been published. First, sensitivity to cisplatin was decreased in ECA-9706 cells highly expressing Homeobox transcription factor NANOG (607937), a gene involved in self-renewal of embryonic stem cells, while the expression of ABCB1 was increased in these cells [164]. On the other hand, *NANOG* knockout suppressed drug resistance to 5-fluorouracil through downregulation of ABCG2 expression and caused G1 arrest by downregulation of CCND1 in ECA-109 cells [165].

Similarly, side population cells in cancers have properties of cancer stem cells and can be resistant to chemotherapy. Signaling pathways involved in cell stemness influence many genes including some ABC transporters. A microarray study identified two genes from ABC family (ABCA5, 612503 and ABCG2) among upregulated genes in a “Tip” side population (with strong Hoechst 33342 dye efflux activity) of EC-9706 cells compared to the non-side population suggesting a possible association of ABC transporters from different gene families with stem cell–specific features [166]. Recently, OE19 and OE33 esophageal adenocarcinoma cell subpopulations surviving 5-fluorouracil treatment exhibited an increase in expression of cancer stem cell markers CD24 (600074) and ABCG2. These cells also exerted increased resistance to apoptosis [167]. Other members of ABC family have been shown related to stemness as well. Apart from stem cell markers CD44 antigen (*CD44*, 107269) and Thymidylate synthase (*TYMS*, 188350), the transcript expression of ABCB1, ABCC2, and ABCC3 was upregulated in OE19 stem cell-like side population compared to non-side population cells [168].

**Figure 4:**
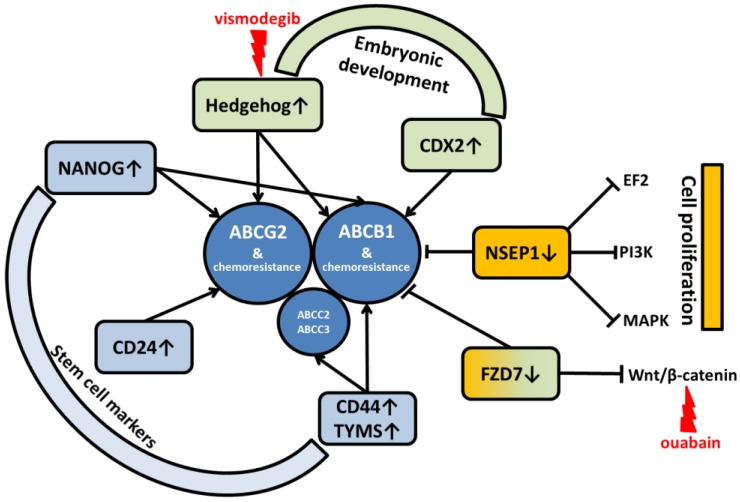
Survey of connections between ABC transporters and signaling pathways in EC and their major roles. Available inhibitors are in red. ↑ = upregulation; ↓ = downregulation; → = stimulation; Ⱶ = inhibition.

It can be concluded that the observed co-segregation of expression of drug efflux ABC transporters with several cancer-driving pathways to which inhibitors are already used in the clinical setting or at least thoroughly investigated (Figure 4) reveals promising applications of combined chemotherapy to counter the multidrug resistance phenomenon. However, data in patients are still missing to evaluate in vivo relevance of such networks.

### 2.5. ABC Transporters-Induced Radiotherapy Resistance

Neoadjuvant chemoradiation represents the current standard of care for localized EC. Several trials have shown that radioresistant cell lines have also increased chemoresistance connected with an increased expression of ABCB1. Radioresistant cell lines have been produced by fractionated irradiation of ESCC tumor cell cultures [149]. This fractionated irradiation of cell lines is similar to fractionated radiotherapy in neoadjuvant chemoradiation and may explain the partial response of the tumors after chemoradiation and early relapse after esophagectomy. Patients not achieving complete response after chemoradiation have significantly worse prognosis compared to those achieving complete response.

Interestingly, in connection with the above purported role of ABC transporters in chemoresistance, Zhang et al. demonstrated that low dose of the radiotherapy (7 Gy) utilizing 0.5 Gy per fraction decreases the level of ABCB1 expression while high dose (28 Gy) utilizing 2 Gy per fraction significantly increases the expression [34]. This finding has clinical importance considering the fact that recommended doses for neoadjuvant chemoradiation of EC range between 41 and 50 Gy [149] and the recommended fractionation per day is usually between 1.8 and 2 Gy. In summary, generally recommended fractionation schedules may significantly increase the chemoresistance of EC tumor cells.

## 3. Conclusions and Future Prospects

EC has very poor prognosis and, therefore, research on predictive and prognostic biomarkers that represent the basis of personalized medicine should be promoted to identify both patients with high chance for response to conservative treatment regimens and those who should rather be managed by experimental therapies in clinical trials. The search for biomarkers may also bring new putative targets for design of future therapeutics.

This review aimed to summarize the data to form a basis for further research on role of membrane ABC transporters in resistance of EC to chemotherapy and, in a more complex way, in disease progression. A more general view on chemoresistance at both pathway and mechanistic level is also presented (Figure 2).

In agreement with the situation in research on other solid tumors (e.g., ovarian cancer [169]), it may be concluded that single genes and protein products as ABCB1, ABCC1, and ABCG2 connected with chemoresistance are well characterized also in EC. However, most data come from in vitro studies using tumor cell models. Studies on cohorts of patients are much scantier and brought conflicting results or results which have not yet been verified in independent patient cohorts. Sadly, other interesting ABCs, e.g., antigen processing ABCB2/ABCB3 or the translation regulating ABCE1, belong to the less studied molecules, similar to the physiologically important ABCs from A, D, and G subfamilies (Table 1). The role of physiological substrates and active ABCs in cancer is being increasingly studied [170,171,172].

Bearing in mind the revolution initiated by the next-generation sequencing technology, the data about role of germline and somatic genetic variability of ABCs in EC etiology, progression and therapy outcome are still very limited. Although epigenetics is thought to be a principal modifier of carcinogenesis [173] and research in this area has been massively accelerated during last decade, studies regarding role of ABC methylome in EC are basically missing and those on miRNAs targeting ABCs are very scarce and incomplete. In contrast to other cancers as breast, colorectal, pancreatic, or ovarian [8,9,10,11], the knowledge concerning the transcript and protein level is not much better (Table 2).

Thus, the authors of this review would like to advocate for focusing of future studies on a more complex assessment of interplay between genetic, epigenetic and phenotypic profiles of larger groups (optimally all genes) of ABC transporters. Preferably, such studies should be complemented with investigation of importance of equilibrium between drug efflux (ABCs) and uptake (SLCs or Copper transporting ATPases-ATPs) for chemoresistance and interplay with other pathways connected with resistance. This task is now technically manageable due to versatility of next-generation sequencing, but still hindered by a highly demanding bioinformatics evaluation of complex and big datasets generated.

Unfortunately, most studies to date investigated either transcript or protein expression of ABCs, but very few compared both together. Complete understanding of the axis gene–expression–protein activity is a prerequisite to any meaningful clinical use of a biomarker or target. Therefore, putative biomarkers from retrospective studies have to be replicated by independent follow up studies and fully functionally characterized in proper in vitro and in vivo models in order to distinguish between causative (both prognostic and targetable) and correlative (just prognostic) biomarkers.

From the clinical perspective, immunohistochemistry is frequently utilized in the clinical setting and can be validated in any pathology unit. Current routine use of immunohistochemistry for subtyping of breast cancer can serve as an example [174] that could be followed in the management of other tumors as well. Moreover, correlation between genotype and gene or protein expression levels may help overcome limitations connected with tumor tissue availability. Biopsies before the neoadjuvant therapy provide limited volume of material and tissues from resection specimens after such therapy may be compromised by developed necrosis and post-radiation changes. Moreover, specimens from recurrent lesions may not always be available for molecular characterization. Therefore, functional germline genetic variants (in the phenotype and clinical context) for pharmacogenomics should be revealed and liquid biopsies as tissue surrogates established where possible.

Finally, recent experimental studies have shown that ABCB1- and ABCG2-mediated chemoresistance may be reversed by simultaneous administration of new targeted therapeutics, e.g., anti-EGFR antibody cetuximab [175], third-generation tyrosine kinase inhibitor active against specifically mutated EGFR, osimertinib [176] or cyclin dependent kinase (CDK4 and CDK6) inhibitor abemaciclib [177]. Although clinical trials in EC patients so far have shown these agents as ineffective (cetuximab, phase III) [178] or the trials are still recruiting (osimertinib, phase II-NCT02465060 and abemaciclib, phase IIh NCT03292250), these agents have potential to downregulate the above ABC transporters in various in vitro or in vivo models and provide a clinically relevant and experimentally intriguing approaches for further progress in this area.

## Figures and Tables

**Table 1:** Characteristics of human ABC transporters.

Gene *	Chromosomal Location *	Gene (bp) *	Protein Name *	Protein (AA) *	Subcellular Localization *	Physiological Substrates/Function *	Examples of Translocated Drugs *	Pathology *
*ABCA1*	9q31.1	147,245	ABC1	2261	PM	cholesterol, phospholipids, sphingomyelin	statins, glyburide, fenofibrate	Tangier disease, hypercholesterolemia
*ABCA2*	9q34.3	21,748	ABC2	2435	lysosome	cholesterol, sterols		Pulmonary surfactant metabolism dysfunction
*ABCA3*	16p13.3	64,869	ABC3	1704	lamellar bodies	phospholipids, sphingomyelin	imatinib, gleevec	Pulmonary surfactant metabolism dysfunction
*ABCA4*	1p22.1	128,315	ABCR	2273	PM	retinoids, phospholipids		Stargardt disease, retinal dystrophy
*ABCA5*	17q24.3	82,934	ABCA5	1642	lysosome	cholesterol	tacrolimus	Gingival fibromatosis with hypertrichosis
*ABCA6*	17q24.2-q24.3	63,904	ABCA6	1617	nucleus, PM	cholesterol, taurocholate		
*ABCA7*	19p13.3	25,472	ABCX	2146	PM	phospholipids		Alzheimer disease
*ABCA8*	17q24.2	88,122	ABCA8	1581	PM	cholesterol		
*ABCA9*	17q24.2	92,956	ABCA9	1624	PM	macrophage lipid homeostasis		
*ABCA10*	17q24.3	97,633	ABCA10	1543	PM	macrophage lipid homeostasis		
*ABCA12*	2q35	207,039	ABCA12	2595	PM, lamellar bodies	phospholipids		Ichthyosis
*ABCA13*	7p12.3	476,050	ABCA13	5058	PM	unknown		
*ABCB1*	7q21.12	209,691	MDR1	1280	PM	phospholipids, sphingolipids, bile salts	anthracyclines, nucleoside analogs, taxanes, vinca alkaloids, statins	
*ABCB2*	6p21.32	8770	TAP1	808	ER	antigen processing and presentation	lapatinib	
*ABCB3*	6p21.32	16,991	TAP2	686	ER	antigen processing and presentation		
*ABCB4*	7q21.12	78,739	MDR3	1286	PM	phospholipids, bile salts	colchicine, anthracyclines, silodosin	Cholecystitis, familial intrahepatic cholestasis
*ABCB5*	7p21.1	161,832	ABCB5	1257	PM	glutathione level modulator	bilastine, dasabuvir, delafloxacin, naldemedine	
*ABCB6*	2q35	9225	MTABC3	842	mitochondrion, lysosome, Golgi	iron homeostasis		Dyschromatosis universalis hereditaria, familial pseudohyperkalemia, microphthalmia
*ABCB7*	Xq13.3	103,561	ABC7	752	mitochondrion	iron homeostasis		Spinocerebellar ataxia, sideroblastic anemia
*ABCB8*	7q36.1	19,361	MABC1	735	mitochondrion	iron homeostasis	doxorubicin	
*ABCB9*	12q24.31	62,942	TAPL	766	lysosome	cytosolic peptides		
*ABCB10*	1q42.13	42,114	MTABC2	738	mitochondrion	iron homeostasis		
*ABCB11*	2q31.1	112,089	BSEP	1321	PM	phospholipids, bile salts	anthracyclines, nucleoside analogs, taxanes, vinca alkaloids, statins	Familial intrahepatic cholestasis
*ABCC1*	16p13.11	193,498	MRP1	1531	PM	phospholipids, bile salts, steroids, cobalamin	irinotecan, anthracyclines, taxanes, vinca alkaloids, statins	Pseudoxanthoma elasticum
*ABCC2*	10q24.2	69,979	MRP2	1545	PM	bilirubin, bile salts	irinotecan, anthracyclines, taxanes, vinca alkaloids, platinum derivatives, nucleoside analogs	Dubin-Johnson syndrome
*ABCC3*	17q21.33	57,476	MRP3	1527	PM	bile salts	anthracyclines, vinca alkaloids, platinum derivatives, nucleoside analogs	
*ABCC4*	13q32.1	281,618	MRP4	1325	PM	bile salts, uric acid	anthracyclines, cyclophosphamide, platinum derivatives, nucleoside analogs	
*ABCC5*	3q27.1	98,082	MRP5	1437	PM	bile salts, folate, glutamate	irinotecan, platinum derivatives, nucleoside analogs, atorvastatin, probenecid, rifampin	
*ABCC6*	16p13.11	74,746	MRP6	1503	PM, nucleus	putative biomineralization modulator	anthracyclines, platinum derivatives, taxanes, vinca alkaloids, etoposide, TKIs	Pseudoxanthoma elasticum, arterial calcifications
*ABCC7*	7q31.2	250,188	CFTR	1480	all compartments	ATP-gated chloride channel	glyburide, ibuprofen, felodipine	Cystic fibrosis, hereditary pancreatitis
*ABCC8*	11p15.1	83,961	SUR1	1581	PM	ATP-sensitive potassium channel regulation	glyburide, tolbutamide, tolazamide.	Neonatal diabetes mellitus, hyperinsulinemic hypoglycemia, familial hyperinsulinism
*ABCC9*	12p12.1	145,141	SUR2	1549	PM	ATP-sensitive potassium channel regulation	glyburide, nicorandil	Dilated cardiomyopathy, hypertrichotic osteochondrodysplasia
*ABCC10*	6p21.1	24,615	MRP7	1492	PM, lysosome	bile salts, steroids, glutathione conjugates	anthracyclines, nucleoside analogs, taxanes, vinca alkaloids, etoposide	
*ABCC11*	16q12.1	80,659	MRP8	1382	PM	bile salts, steroids, cyclic nucleotides, glutathione conjugates, folate, taurocholate	estrogens, fluorouracil, methotrexate, probenecid, indomethacin	
*ABCC12*	16q12.1	73,046	MRP9	1359	PM	unknown		
*ABCD1*	Xq28	19,912	ALD	745	peroxisome	very long chain fatty acids	ergocalciferol	Adrenoleukodystrophy, Addison’s disease
*ABCD2*	12q12	101,296	ALDL1	740	peroxisome	very long chain fatty acids		
*ABCD3*	1p21.3	100,581	PXMP1	659	peroxisome	very long and long chain fatty acids, branched-chain fatty acids, bile acid intermediates		Bile acid synthesis defect
*ABCD4*	14q24.3	17,788	PXMP1L	606	lysosome	cobalamin		Methylmalonic aciduria and homocystinuria
*ABCE1*	4q31.21	31,593	RNASEL1	599	mitochondrion, cytosol	translation and ribosome biogenesis		
*ABCF1*	6p21.33	25,804	ABC50	845	nucleus, cytosol	translation initiation		
*ABCF2*	7q36.1	19,545	ABC28	623	mitochondrion, cytosol	cell volume regulation		
*ABCF3*	3q27.1	8430	ABCF3	709	nucleus, cytosol	putative translational regulation and apoptosis		
*ABCG1*	21q22.3	104,699	ABC8	678	ER, PM	cholesterol, phospholipids, sterols	irinotecan, fluorouracil, leucovorin	Tangier disease, sitosterolemia
*ABCG2*	4q22.1	141,154	BCRP	655	PM, nucleus	sterols, heme	anthracyclines, platinum derivatives, nucloside analogs, taxanes, vinca alkaloids, etoposide	Tangier disease, sitosterolemia
*ABCG4*	11q23.3	13,655	WHITE2	646	ER, PM	lipids, cholesterol, sterols		
*ABCG5*	2p21	32,711	sterolin 1	651	PM	cholesterol, sterols	ezetimibe	Sitosterolemia
*ABCG8*	2p21	51,236	sterolin 2	673	PM	cholesterol, sterols	ezetimibe, atorvastatin	sitosterolemia, atherosclerosis

Footnotes: * The table was prepared by help of OMIM, GeneCards, ClinVar and PubMed databases; Unknown features or no data in grey; Abbreviations: AA, amino acids, bp, base pairs, ER, endoplasmic reticulum, PM, plasma membrane, TKIs, tyrosine kinase inhibitors.

**Table 2:** Studies on associations between human ABC transporters and esophageal carcinoma in patients.

Level	Gene	Factor	Patients (*N*)	Association	Significance	Reference
Gene germline	*ABCB1*	rs1045642-T allele	210 ESCC and EAC NACRT-treated	longer overall survival after cisplatin	*p* = 0.030	[17]
	*ABCB1*	rs1045642	31 ESCC	no effect on response and survival	NS	[19]
	*ABCB1*	rs1045642-T allele	116 ESCC and 146 EAC NACRT-treated	longer overall survival	*p* = 0.048	[14]
	*ABCB1*	rs1045642	meta-analysis	putative biomarker of recurrence and survival	LOE IV ^#^	[21]
	*ABCB1* **	rs2032582 **	62 ESCC and 62 EAC NACRT-treated	relapse-free and cancer-free survival	*p* < 0.001 **	[18]
	*TAP1*	rs1135216-G allele	361 ESCC and 66 controls	higher risk of ESCC	0.018	[22]
	*TAP1*	rs1135216-AG heterozygote	200 ESCC and 100 controls	higher risk of ESCC	*p* = 0.035	[23]
	*TAP2*	rs1800454-AA homozygote	265 ESCC and 357 controls	higher risk of ESCC	*p* = 0.023	[24]
	*ABCC2* *	rs17222723 *	116 ESCC and EAC NACRT-treated	response	*p* = 0.002 *	[25]
	*ABCC3* *	rs2277624 *	116 ESCC and EAC NACRT-treated	response	*p* = 0.002 *	[25]
Gene somatic	*ABCC4*	presence of CNV	1048 Chinese Han subjects	higher ESCC risk and poor overall survival	*p* = 0.001	[26]
	all ABCs	SNV	2046 ESCC and 568 EAC	unknown	unknown	[27]
Epigenetics-miRNA	ABCB1	high miR-296	25 ESCC	poor survival	*p* < 0.05	[28]
	ABCB1	high miR-483 and miR-214	104 ESCC	poor survival	*p* < 0.05	[29]
Transcript	ABCB1	expression	46 ESCC	no prognostic role	NS	[30]
	ABCB1	expression	31 EAC NACRT-treated	no prognostic role	NS	[31]
	ABCB1	high expression	40 EAC NACRT-treated	no prognostic role	NS	[32]
	ABCB1	expression	310 ESCC	poor overall survival	*p* = 0.014	[33]
	ABCB1	expression	54 unspecified EC derived cell lines	↑ after high dose ↓ after low dose radiotherapy	*p* < 0.05	[34]
	ABCC1	high expression	38 EAC NACRT-treated	longer overall survival and response	*p* = 0.017/*p* = 0.007	[35]
	ABCC1	low expression	31 EAC NACRT-treated	response to NACRT	*p* = 0.041	[31]
	ABCC1	high expression	40 EAC NACRT-treated	no prognostic role	NS	[32]
	ABCC2	high expression	42 ESCC NACRT-treated	poor response to NACT	*p* = 0.003	[36]
	ABCG2	high expression	33 ESCC	poor survival	*p* = 0.017	[37]
Protein	ABCB1	high expression	118 EAC and ESCC NACRT-treated	poor cancer-free survival	*p* = 0.05	[38]
	ABCB2	expression	143 ESCC	correlates with tumor grade and metastasis	*p* < 0.05	[39]
	ABCC1	high expression	40 EAC NACRT-treated	poor response to chemotherapy	*p* = 0.036	[32]
	ABCC1	expression	116 ESCC	no prognostic role	NS	[40]
	ABCC1	expression	829 ESCC	no prognostic role	NS	[41]
	ABCC2	high expression	582 ESCC	correlates with tumor grade	*p* < 0.01	[42]
	ABCC2	expression	81 ESCC	poor overall survival and response to NACRT	*p* = 0.027/*p* = 0.003	[36]
	ABCC3	IgA autoantibodies	114 ESCC and 226 controls	diagnostic and predictive biomarker	*p* < 0.001	[43]
	ABCE1	expression	112 ESCC	correlates with ESCC grade and stage	*p* < 0.001	[44]
	ABCG2	expression	100 ESCC	poor survival	*p* = 0.009	[37]
	ABCG2	high expression	110 ESCC	poor overall survival	*p* = 0.005	[45]

Footnotes: * As part of five-gene (*ABCC2*, *ABCC3*, *CYP2A6*, *PPARG*, and *SLC7A8*) predictive panel; ** as part of five-polymorphism (*ABCB1*, *MTHFR*, *GSTP1*, and two in *XPC)* panel; ^#^ Cumulative Level Of Evidence [46]. Abbreviations: CNV, copy number variation; EAC, esophageal adenocarcinoma; ESCC, esophageal squamous cell carcinoma; EC, esophageal carcinoma of unspecified type; NACRT, neoadjuvant chemo-radiotherapy; NS, non-significant; SNV, single nucleotide variation.

## Supplementary Materials

Supplementary Materials can be found at http://www.mdpi.com/1422-0067/19/3/868/s1. Table S1: (A) Somatic mutations in coding sequences of ABC transporter genes in esophageal cancer from COSMIC database; (B) Somatic copy number variations of ABC transporter genes in esophageal cancer from COSMIC database; (C) Differences in gene expression of ABC transporter genes in esophageal cancer from COSMIC database; Table S2: Role of ABC transporter genes in cell models of esophageal cancer.
